# Supplementary material for: Metagenomic Analysis of a Continuous-Flow Aerobic Granulation System for Wastewater Treatment
Source: Microorganisms. 2023 Sep 15;11(9):2328. doi: 10.3390/microorganisms11092328 (PMC10535324; doi:10.3390/microorganisms11092328)
Supplement: Supplementary file 1 [file microorganisms-11-02328-s001.zip › microorganisms-2558617-supplementary.pdf]

Metagenomic Analysis of a Continuous-Flow Aerobic Granulation System for Wastewater Treatment

Alison T. Gomeiz [1], Yewei Sun [2], Aaron Newborn [1], Zhi-Wu Wang [2], Bob Angelotti [3] and Benoit Van Aken [1,*]

[1] Department of Chemistry and Biochemistry, George Mason University, 4400 University Dr, Fairfax, VA 22030, USA; agomeiz@gmu.edu (A.T.G.); anewborn@gmu.edu (A.N.)
[2] Occoquan Laboratory, Biological Systems Engineering, Virginia Tech, 1230 Washington St SW,
Blacksburg, VA 24061, USA; ysun@hazenandsawyer.com (Y.S.); wzw@vt.edu (Z.-W.W.)
[3] Upper Occoquan Service Authority, 14631 Compton Rd, Centreville, VA 20121, USA; bob.angelotti@uosa.org
* Correspondence: bvanaken@gmu.edu

Supplementary Information

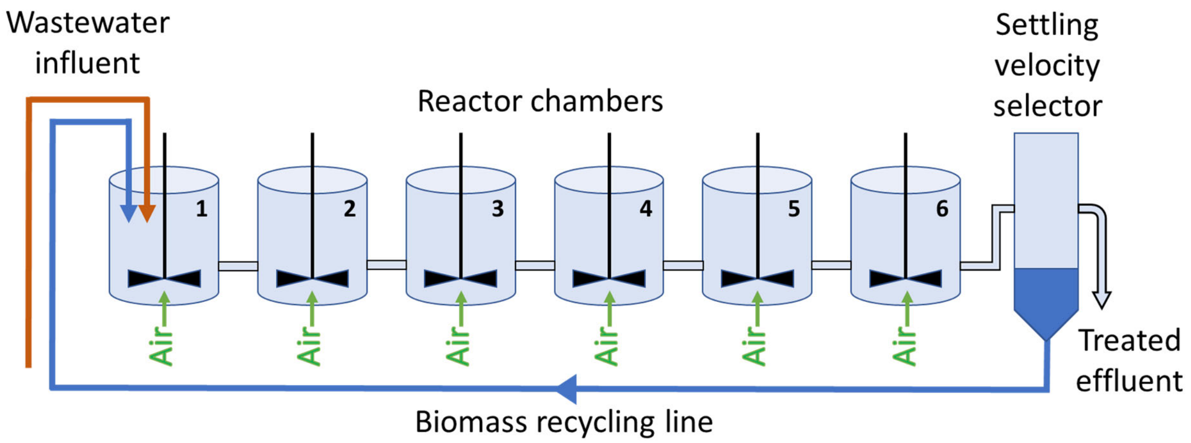

Figure S1. Schematic representation of one of the 6-chamber simulated PFR used in this study."

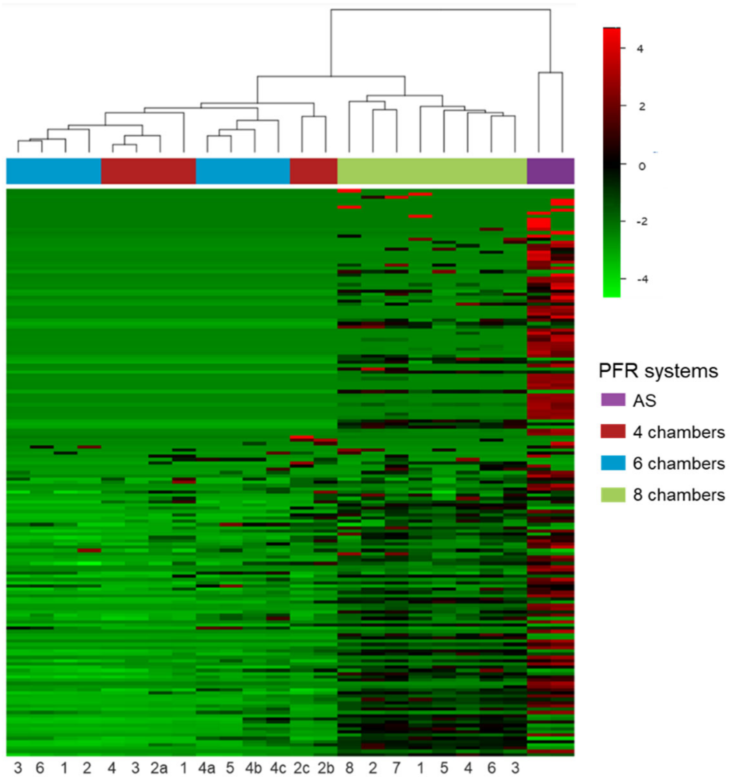

Figure S2. Heatmap showing the relative bacterial abundances between the three PFRs and AS at the order taxonomic level: AS control (purple), 4 chambers (red), 6 chambers (blue), and 8 chambers (green). Orders are plotted by decreasing relative abundance. The colors in the heatmap reflect the normalized abundance of each order.
